# Effects of a pulmonary rehabilitation program on physical capacity, peripheral muscle function and inflammatory markers in asthmatic children and adolescents: study protocol for a randomized controlled trial

**DOI:** 10.1186/s13063-015-0876-x

**Published:** 2015-08-13

**Authors:** Mariana Mazzuca Reimberg, Rejane Agnelo Silva Castro, Jessyca Pachi Rodrigues Selman, Aline Santos Meneses, Fabiano Politti, Márcia Carvalho Mallozi, Gustavo Falbo Wandalsen, Dirceu Solé, Kátia De Angelis, Simone Dal Corso, Fernanda Cordoba Lanza

**Affiliations:** Master Degree in progress at Postgraduate Program in Rehabilitation Sciences, Universidade Nove de Julho - UNINOVE, Sao Paulo/SP, Brazil; Affiliated with the Postgraduate Program in Rehabilitation Sciences, Universidade Nove de Julho - UNINOVE, Sao Paulo/SP, Brazil; Division of Allergy, Clinical Immunology and Rheumatology, Department of Pediatrics, Federal University of Sao Paulo, Sao Paulo/SP, Brazil; Post Graduation Department, Universidade Nove de Julho - UNINOVE, Vergueiro St, 235/249, São Paulo, SP 01504-001 Brazil

**Keywords:** Asthma, Physical training, Aerobic capacity, Quality of life

## Abstract

**Background:**

Individuals with chronic lung disease are more susceptible to present reduction in exercise tolerance and muscles strength not only due to pulmonary limitations but also due systemic repercussions of the pulmonary disease.

The aim of this study is to assess the physical capacity, peripheral muscle function, physical activity in daily life, and the inflammatory markers in children and adolescents with asthma after pulmonary rehabilitation program.

**Method:**

This is a study protocol of randomized controlled trial in asthmatic patients between 6 to 18 years old. The assessments will be conducted in three different days and will be performed at the beginning and at the end of the protocol. First visit: quality of life questionnaire, asthma control questionnaire, pre- and post-bronchodilator spirometry (400 μcg salbutamol), inflammatory assessment (blood collection), and cardiopulmonary exercise test on a cycle ergometer to determine aerobic capacity. Second visit: assessment of strength and endurance of the quadriceps femoris and biceps brachii muscles with concomitant electromyography to assess peripheral muscle strength. Third visit: incremental shuttle walk test (ISWT) and accelerometer to evaluate functional capacity and physical activity in daily life during 7 days. Then, the volunteers will be randomized to receive pulmonary rehabilitation program (intervention group) or chest physiotherapy + stretching exercises (control group). Both groups will have a supervised session, twice a week, each session will have 60 minutes duration, with minimum interval of 24 hours, for a period of 8 weeks. Intervention group: aerobic training (35 minutes) intensity between 60 to 80 % of the maximum workload of cardiopulmonary exercise testing or of ISWT; strength muscle training will be applied to the quadriceps femoris, biceps brachii and deltoid muscles (intensity: 40 to 70 % of maximal repetition, 3 x 8 repetition); finally the oral high-frequency oscillation device (Flutter®) will be used for 5 minutes. The control group: oral high-frequency oscillation device (Flutter®) for 10 minutes followed by the stretching of upper and lower limbs for 40 minutes. It is expected to observe the improvement in aerobic capacity, physical activity in daily life, muscle strength and quality of life of patients in the intervention group, and reduction in inflammatory markers.

**Trial registration:**

Clinical Trial Number: NCT02383069. Data of registration: 03/03/2015

## Background

Asthma is one of the main chronic diseases in childhood. It is characterized by inflammation of the airways [1]. Individuals with chronic lung disease are more susceptible to present reduction in exercise tolerance, not only due to ventilatory constraints, but also due to systemic manifestations of pulmonary disease [2]. Thus, the more severe and the less controlled the disease, the higher the dyspnea and fatigue reported by patients. In general, the obstruction increases the airways resistance, hindering the physiological ventilatory response during physical exertion and leading to dyspnea [2]. This in turn leads to the patient having a more sedentary lifestyle, predisposing them to early fatigue and exercise intolerance.

Cardiopulmonary exercise testing (CPET) is the gold standard for determining exercise intolerance [3, 4]. As it is a high-cost assessment, few studies have used CPET as a method to evaluate asthmatic children and adolescents [5–9]. Villa and colleagues [6] described the reduction of oxygen uptake (VO_2peak_ ) in patients with moderate/severe asthma when compared to the control group. The same was observed by an English group that found VO_2max_ reduction in the asthmatic group [7, 8]. Those authors proposed that asthma severity could be an important factor in determining aerobic capacity. Some limitations were observed in those mentioned studies, such as not measuring asthma control (a factor that may influence the patient’s condition), the small number of assessed individuals, and not determining the corticosteroid dose used by patients with asthma.

Clinical field tests are a less expensive alternative to CPET in determining functional capacity. The incremental shuttle walking test (ISWT) is a simple and inexpensive test described by Singh and colleagues [10]. Several authors using ISWT assessment found a reduction in the functional capacity of adults with chronic lung disease and its association with lung function and quality of life [11–13]. To our knowledge, only Ahmaidi and colleagues [14] have used the run shuttle walk test in pediatric patients with asthma to determine their functional capacity by comparing with CPET.

Reduction in peripheral muscle strength has been described in patients with cystic fibrosis [15] and asthma [6], and the hypothesis for that is that it is due to sedentary lifestyle, the chronic use of medication, and systemic inflammation. To our knowledge, there are no studies evaluating muscle strength and peripheral muscle endurance by using electromyography in asthmatic children.

Some studies have had interesting results in cardiopulmonary conditions, quality of life, and reduction in the number of hospitalizations after a pulmonary rehabilitation program in asthmatic children and adolescents [14, 16–23]. Wanrooij and colleagues [24] carried out a systematic review of physical training with asthmatic children and adolescents, and concluded that physical activity should be recommended to this population, although some issues have not yet been clarified due to limitations in the clinical trials. The control of the disease has not been addressed by specific questionnaires in any study. The assessment of inflammatory markers has rarely been addressed [21], neither has quality of life after physical training [20, 21].

Hence, the current study aims to assess physical capacity, peripheral muscle function, physical activity in daily life, quality of life, and inflammatory markers in children and adolescents with asthma, after undergoing a pulmonary rehabilitation program.

## Methods

### Study design

This is a randomized clinical trial to be conducted in the pulmonary rehabilitation laboratory at University Nove de Julho. Clinically diagnosed asthmatic patients will be recruited in the Allergy, Clinical Immunology and Rheumatology Clinic at the Department of Pediatrics of Sao Paulo School of Medicine, Federal University of Sao Paulo. The individuals will be enrolled in the study after their legal guardians have read, agreed to, and signed the informed consent form. The current project was approved by the Associacao Educacional Nove de Julho Ethical Committee, Sao Paulo, Brazil, number 738192/2014.

The study will include patients diagnosed with asthma, between 6 and 18 years old, who are under medical treatment and disease control according to the Global Initiative for Asthma (GINA) criteria [1]. Those who fail to carry out the protocol evaluations, who interrupt medical care and/or drug-based treatment, who present with acute lung infection, other chronic lung diseases, or other comorbidities (neuropathies, heart disease), and those who miss more than 20 % of the rehabilitation sessions will be excluded from the study.

### Outcomes

The primary outcome will be the physical capacity assessed by the cardiopulmonary exercise testing and ISWT. The secondary outcomes will be quality of life, peripheral muscle strength, and inflammatory markers.

### Assessments

#### Questionnaires

The pediatric asthma quality of life questionnaire (PAQLq) [25] will be used. It is composed of 23 questions divided into three domains: physical activity limitations (5 questions), symptoms (10 questions) and emotions (8 questions). The responses will be measured using a 7-point scale, according to which 1 indicates the maximum loss and 7 indicates no loss.

The asthma control questionnaire (ACT or C-ACT) will be applied according to the patient's age [26] to determine whether asthma is controlled. The ACT consists of five questions. The scores of each question range between 1 and 5 points. The questionnaire minimum score is 5 points and the maximum score is 25 points. The C-ACT, which will be applied to children under 11 years and 12 months, consists of seven questions, four of them answered by the children and three by the parents/guardians, with a minimum score of 0 and maximum of 27.

#### Pulmonary function

Spirometry tests will be performed using ULTIMA CPX equipment (MedGraphics Corporation®, St Paul, MN, USA). The technical procedures, acceptance criteria and reproducibility will be adopted according to recommendations [27]. All patients will perform the maneuvers post bronchodilation (salbutamol 400 μcg). The following variables will be recorded: forced vital capacity (FVC), forced expiratory volume at the first second (FEV_1_), FEV_1_/FVC ratio and forced expiratory flow (FEF25-75) [28].

#### Cardiopulmonary exercise testing

The exercise test will be performed in an electromagnetic braking cycle ergometer (Corival®, LODE BV Medical Technology, Groningen, Netherlands) connected to a system composed of gas exchange and ventilatory variables being analyzed breath by breath (Breeze CardiO_2_ System® microcomputer; Medical Graphics Corporation-MGC, St Paul, MN, USA). After 2 minutes of freewheel load, the load will be increased (5 to 20 watts/minute) and the test will be limited between 8 and 12 minutes [29, 30]. The following measurements will be analyzed: oxygen consumption (VO_2_, mL min^−1^) and carbon dioxide production (VCO_2_, mL.min), minute ventilation (V_E_, L/minute), tidal volume (TV) (mL), respiratory rate (f) (respirations/minute), and ventilatory equivalents for O_2_ and CO_2_ (V_E_/VO_2_, V_E_/VCO_2_). The heart rate (HR), pulse oxyhemoglobin saturation (SpO_2_) will be continuously recorded. Blood pressure (BP) will be measured every 2 minutes of exercise. Dyspnea (Borg D) and lower limb fatigue (Borg LL) perception scores will be assessed by using the modified Borg scale [31], with the patient at rest and immediately after the exercise cessation. The test will be interrupted by the child or adolescent due to intolerable dyspnoea and/or fatigue making it impossible to progress the workload. On the other hand, the test will be stopped if SpO_2_ is ≤82 %, or if patients have cardiac arrhythmias or abnormal blood pressure response.

#### Incremental shuttle walking test (ISWT)

The ISWT will be held in a 10-m-long corridor according to the original description [10]. It is a test externally cadenced by an audible signal, in which the speed increases every minute ranging from 1.79 to 10.2 Km/h. The test comes to the end when the patient is not able to reach the cone two consecutive times, or the patient needs to stop the test due to fatigue or breathlessness, or SpO_2_ falls below 82 %. The tests will be performed twice on the same day, with a 30-minute break between them. Heart rate, blood pressure, and Borg fatigue and Borg dyspnea will be evaluated at the beginning and at the end of the test. The total test time and the distance walked by the patient will be recorded at the end of the test.

#### Physical activity in daily life

Each patient will be monitored by an ActiGraph accelerometer (GT3X) throughout seven consecutive days [32]. The accelerometer will be firmly placed on the patient’s hip (pelvic girdle), and it will be removed just for sleeping, bathing and swimming. Each individual or his/her guardian will be given a form for the daily descriptions of unusual activities performed while using the device. The form must be completed at the end of the day. The number of steps will be recorded for analysis.

#### Skeletal muscle function assessment

The maximum isometric voluntary contraction (MIVC) of the quadriceps femoris (QF) muscle will be obtained by having the individuals sit on a leg extension machine (Carci®, São Paulo, Brazil) with their knees positioned at 60 ° flexion. An inelastic cable connected to a load cell and adapted to an anklet (EMG System model EMG800C, São José dos Campos, Brazil) will be positioned perpendicular to the non-dominant lower limb. This cell will capture the muscle tension developed during knee extension MIVC and it will record the force (Kgf).

The MIVC of the biceps brachii (BB) muscle will be obtained by having the individuals sit on a chair. The same equipment will be connected to a load cell and it will be positioned perpendicular to the non-dominant upper limb. This cell will capture the muscle tension developed during the MIVC of elbow flexion and record it on the computer.

Three replicates will be held for 5 seconds, with a 1-minute rest interval between all the measures [33]. The greatest value of these three contractions of QF and BB will be considered as the MIVC.

After a resting period of five minutes, the isometric endurance test (IET) of the QF will be evaluated by the isometric endurance time at 60 % of the MIVC until the limit of tolerance (Tlim). The isometric endurance test will be finished when a 10 % drop of the produced force occurs. The same procedure will be used to verify the endurance of the IET by BB. All measurements will be performed with visual feedback on the computer screen. Dyspnea and leg fatigue will be evaluated before and immediately after the test by the modified Borg scale. This test will take approximately 15 minutes.

#### Surface electromyography (*s*EMG)

The surface electromyographic (sEMG) will be recorded in the dominant upper and lower limb. Active bipolar surface electrodes will be positioned in the belly of the evaluated muscles (RF and BB) as previously described [34]. The *s*EMG (EMG System, model EMG800C, São José dos Campos, Brazil) will be recorded using a 4-channel device (EMG System do Brazil Ltda®), with a band pass filter with cutoff frequencies of 20 to 500 Hz, an amplifier gain of 1000, and a common rejection mode ratio >100 dB. All data will be acquired and processed using a 12-bit analog-to-digital converter, with a sampling frequency of 2 kHz. The obtained signal will be amplified and converted to digital format for data recording and analysis.

The sEMG signal will be divided into 1-second windows and the root mean square (RMS) and median frequency (MDF) of the power spectrum will be calculated for each window. Five consecutive values over RMS time and MDF will be averaged to obtain mean values corresponding to 0, 25, 50, 75, and 100 % of the endurance time. All EMG signals will be processed performing specific routines carried out in the Matlab program, R2010b (The MathWorks Inc., Natick, MA, USA).

#### Inflammatory markers

Blood IL-4, IL-5, IL-10, IL-13, PCR, and TNFα will be assessed [35]. The plasma will be centrifuged for 10 minutes at 3,000 rpm in a centrifuge refrigerated at 0 to 4 °C (Eppendorf, 5804-R), and the supernatant will be frozen in a freezer at −80°C for dosing. Dosing of cytokines and TNFα will be performed in plasma, in microplates (96 wells) sensitized with the antibody to the protein of interest, adhered to the wall of the plate wells by an immunoadsorbent substrate. The following human-specific kits will be used: human TNFα ELISA kit, human IL-4, IL-5 ELISA kit, human IL-10, *and* human IL-13 ELISA kit.

### Protocol

**Fig 1:**
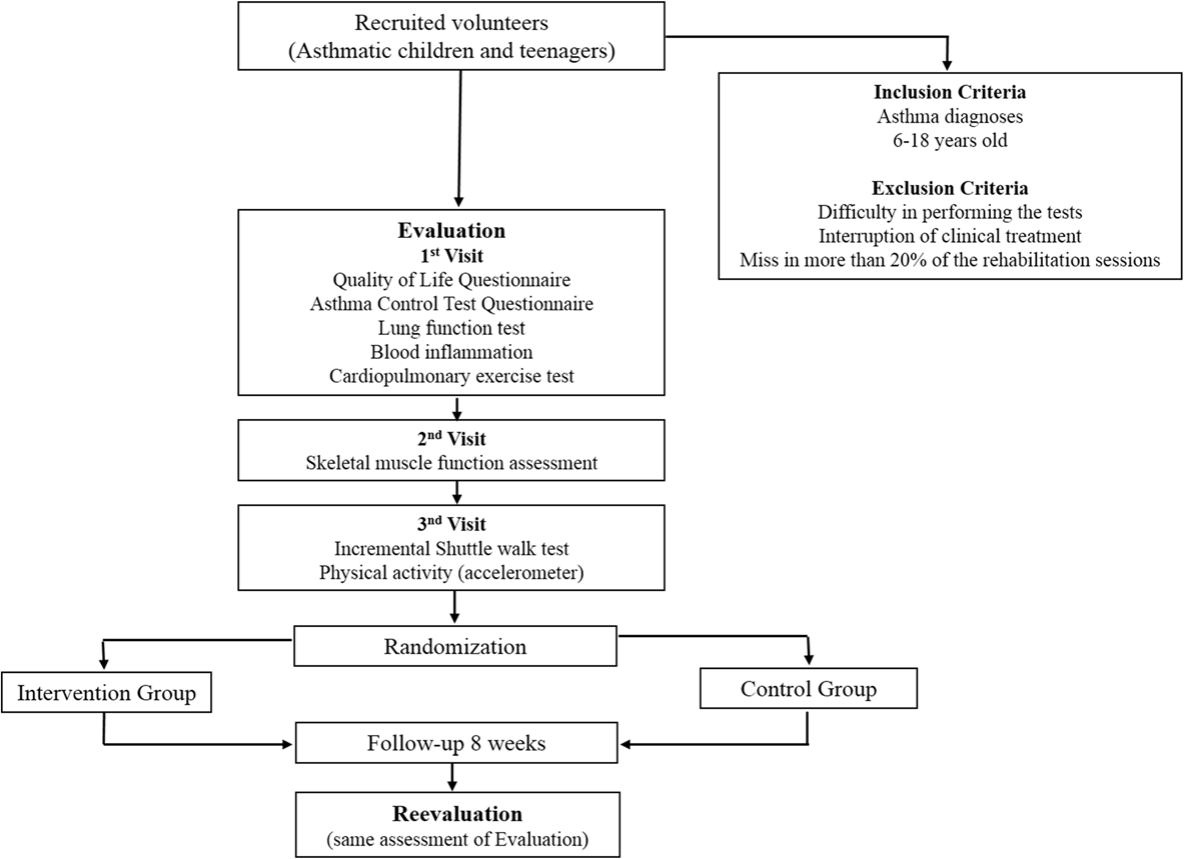
Flow of patients through the study

The assessments will be conducted in three visits, as described below (Fig. 1).

Visit 1: quality of life questionnaire, asthma control questionnaire, pre- and post-bronchodilator spirometry (400 μcg salbutamol), inflammatory assessment (blood collection), and cardiopulmonary exercise test on a cycle ergometer.

Visit 2: Assessment of strength and endurance of the quadriceps femoris and biceps brachii muscles with surface electromyography.

Visit 3: ISWT and an accelerometer will be used to assess physical activity in daily life.

The volunteers will be randomized to receive a pulmonary rehabilitation program (intervention group) or chest physiotherapy plus stretching exercises (control group), as described below by the website randomization.com.

### Intervention group

The intervention group will have a supervised rehabilitation program held twice a week. Each session will be 60 minutes in duration, with a minimum interval of 24 hours, for a period of 8 weeks. Each session will consist of three parts: aerobic training, strength muscle training, and chest physiotherapy. The aerobic training will be held for 35 minutes (10 minutes of warm up, 20 minutes on target load, and 5 minutes of cooling down) with an initial intensity of 60 % of the maximum load obtained in the maximal cardiopulmonary exercise testing or in the ISWT. The intensity will be gradually increased up to 80 %, so that fatigue or dyspnea values are kept between 4 and 6, according to the modified Borg scale [31]. When necessary, supplemental oxygen will be supplied during training to keep oxygen saturation greater than 92 %. Strength muscle training will be applied to the quadriceps femoris, biceps brachii, and deltoid muscles for 15 minutes. The intensity will be 40 to 70 % of maximal repetition (1 MR) in three sets of eight repetitions. Finally, for chest physiotherapy, the oral high-frequency oscillation device (Flutter®) will be used for 5 minutes. The volunteers in this group will receive a 200-μcg bronchodilator before starting the session each day.

### Control group

The control group will have chest physiotherapy and stretching exercises twice a week. Each session will be 60 minutes, with a minimum interval of 24 hours, for a period of 8 weeks. For each session, the oral high-frequency oscillation device (Flutter®) will be used for 10 minutes, 5 minutes in each lateral decubitus position, followed by stretching of the upper and lower limbs for 40 minutes. All exercises will be active, performed in sitting and lying positions without increasing the heart rate. The remaining 10 minutes will be used to discuss doubts about the disease and the use of the booklet.

Both groups will be oriented about the disease (proper use of medication, use of peak flow, triggering factors for crisis). On the date of the initial evaluation, they will take home a booklet with the main information about the given guidelines. All volunteers will be reevaluated after 8 weeks, when the same initial assessments will be performed again.

### Statistical analysis

The sample size was based on the run shuttle walk test levels and on the maximum load achieved in CPET [14]. Considering the stages of the shuttle, and assuming an alpha risk of 5 % and beta risk of 80 %, standard deviation of 1.6, and a difference in magnitude of 2.0 levels before and after the pulmonary rehabilitation program [14], the sample size needed was 27 patients in each group. Considering the load (W) achieved in the cardiopulmonary exercise testing, with a −3 ± 2 W difference pre- and post-intervention in the control group, and of 16 ± 7 [20] in the intervention group, with an alpha risk of 5 % and a beta risk of 95 %, the sample size needed was 10 patients in each group. Therefore, considering potential losses, 30 asthmatic patients will be recruited for each group, requiring a total of 66 volunteers.

The data normality will be analyzed using the Kolmogorov-Smirnov test. Parametric data will be represented as means (SD) and nonparametric data as medians (IQR (25th−75th percentiles ). Comparisons between groups and time (pre- and post-rehabilitation) will be analyzed by two-way analysis of variance (ANOVA), with the Bonferroni test post hoc, or by the Kruskal Wallis test, depending on the data adherence or non-adherence to the Gaussian curve. Correlation between variables will be analyzed by Pearson or Spearman correlation according to the data distribution. Three-way repeated measures ANOVA group vs endurance (0–100 % endurance time, 25 % increments time) vs time (pre- and post-) will be used to assess the dependency of the EMG variables and will followed by post hoc Student–Newman–Keuls pairwise comparisons, when appropriate. The value of *P* <0.05 will be set as significant. SPSS version 13 (Chicago, IL, USA) will be the statistical software used in the study.

## Discussion

Asthma is the most common chronic lung disease in the pediatric population. The dyspnea sensation during exercise in these patients is secondary to increases in airway resistance, pulmonary hyperinflation, and hypoxemia observed in severely affected patients [2]. Some studies have described a reduction in exercise capacity in asthmatic pediatric patients [5–9]. The main hypothesis is that this is due to the deconditioning of the patient, which is a result of the pathogenesis of asthma. The more severely affected the patient, the greater the deconditioning. However, some issues have not been widely addressed to justify the reduction in aerobic capacity: the control of asthma, the severity according to the Asthma Consensus [1], the amount of medication, and the level of physical activity.

In addition to the decrease in aerobic capacity, a reduction in peripheral muscle strength is observed in chronic lung disease [6, 15, 36]. Villa et al. [6] described a reduction in skeletal muscle function in asthmatic patients. They observed that endurance was lower in the quadriceps muscle of severe asthmatic patients compared to moderate and intermittent disease. The hypotheses about this muscle restriction are based on the chronic use of corticosteroids, sedentary lifestyles, and the reduction in aerobic capacity, but the literature about muscle strength and endurance in asthmatic pediatric patients is scarce. More studies must be done to answer this question, including assessment of the amount of medication and the level of physical activity.

Physical activity may be evaluated by subjective methods or a questionnaire, but the cognitive and physiological changes can be difficult in this evaluation for a pediatric population. It can also be evaluated by objective methods such as an accelerometer, which is the gold standard [37]. The correlation between physical activity and lung function was first addressed by Pitta et al. [38] in adult patients with chronic obstructive pulmonary disease. To our knowledge, physical activity evaluated by accelerometer in pediatric asthmatic patients has only been reported by Souza et al. [39]. The group did not observe differences in the number of steps between the asthma and control groups, but some asthmatic children were overweight, and the group did not evaluate teenagers.

Pulmonary rehabilitation is recommended for patients with chronic lung disease with reduction in aerobic capacity and muscle strength [40]. It was described in two systematic reviews of asthmatic pediatric patients that exercise training reduces the risk of exacerbation, exercise-induced bronchospasm, and increases in quality of life [24, 41]. However, some issues are not clear about pulmonary rehabilitation in the pediatric population. There are different intensities of training described, the control of asthma has to be well-addressed, muscle strength and endurance are poorly evaluated, the severity of disease is not described as determined by consensus [1], physical activity is not usually measured by a gold standard, and protocols that evaluate inflammatory markers, such as cytokines are rare [42].

The current study hopes to determine the benefits of pulmonary rehabilitation in children and adolescents diagnosed with asthma, such as improvement in functional capacity, physical activity in daily life, muscle strength, quality of life and inflammatory markers. Thus, we will have greater support in the use of physical exercise in the asthmatic pediatric population.

## Trial status

Patient are been recruited at the time of submission.

## Abbreviations

ACT: astha control test
ANOVA: analysis of variance
BB: biceps brachii
BP: blood pressure
C-ACT: childhood asthma control test
CPET: cardiopulmonary exercise testing
ELISA: enzyme-linked immunosorbent assay
f: respiratory rate
FEF25-75: forced expiratory flow
FEV1: forced expiratory volume at the first second
FVC: forced vital capacity
GINA: Global Initiative for Asthma
HR: heart rate
IET: isometric endurance test
IL: interleukin
ISWT: incremental shuttle walk test
MDF: median frequency
MIVC: maximum isometric voluntary contraction
MR: maximal repetition
PAQLq: pediatric asthma quality of life questionnaire
QF: quadriceps femoris
RMS: root mean square
sEMG: surface electromyographic
SpO_2_: pulse oxyhemoglobin saturation
TNF: tumor necrosis factor
TV: tidal volume
VCO_2_: carbon dioxide production
VE/VCO_2_: ventilatory equivalents for CO_2_
VE/VO_2_: ventilatory equivalents for O_2_
VE: minute ventilation
VO_2_: oxygen consumption
